# Supplementary material for: Blood RNA signatures outperform CRP triage of tuberculosis lymphadenitis and pericarditis
Source: medRxiv. 2024 Jun 21:2024.06.21.24309099. Preprint. [Version 1] doi: 10.1101/2024.06.21.24309099 (PMC11213046; doi:10.1101/2024.06.21.24309099)
Supplement: Supplement 1 [file media-1.pdf]

Supplementary data

Contents

Supplementary data 1

Contents 1

Supplementary Figure 1 2

Testing algorithm for patients with possible TB lymphadenitis or pericarditis 2

Supplementary Figure 2 3

Principal component analysis of Nanostring results from reference RNA sample. 3

Supplementary Figure 3 4

Distribution of gene expression data stratified by Nanostring codeset batch 4

Supplementary Figure 4 5

Distributions of TB genes after ComBat 5

Supplementary Figure 5 6

Sample size calculations. 6

Supplementary Figure 6 7

Blood RNA biomarker discrimination of primary reference standard for lymph node TB. 7

Supplementary Figure 7 8

Blood RNA biomarker discrimination of primary reference standard for pericardial TB. 8

Supplementary Figure 8 9

Blood RNA biomarker discrimination of lymph node and pericardial TB including trace Ultra results 9

Supplementary Figure 9 10

Blood RNA biomarker discrimination of lymph node and pericardial TB including TB treatment initiation. 10

Supplementary Figure 10 11

Blood RNA biomarker discrimination of lymph node and pericardial TB including including cytology and histology diagnosis of TB. 11

Supplementary Figure 11 12

Blood RNA biomarker discrimination of primary reference standard for lymph node and pericardial TB using raw Nanostring data without batch correction. 12

Supplementary Table 1 13

Baseline characteristics of the study cohort, stratified by inclusion 13

Supplementary Table 2 14

Blood RNA biomarker and CRP performance metrics for discrimination of primary reference standard in lymph node and pericardial TB, using alternative cut-offs. 14

Supplementary Table 3 15

Blood RNA biomarker and CRP performance metrics for discrimination of primary reference standard in lymph node TB 15

Supplementary Table 4 16

Blood RNA biomarker and CRP performance metrics for discrimination of primary reference standard in pericardial TB 16

Supplementary Table 5 17

Blood RNA biomarker and CRP performance metrics for discrimination of TB status, stratified by selected subgroups. 17

Supplementary Figure 1

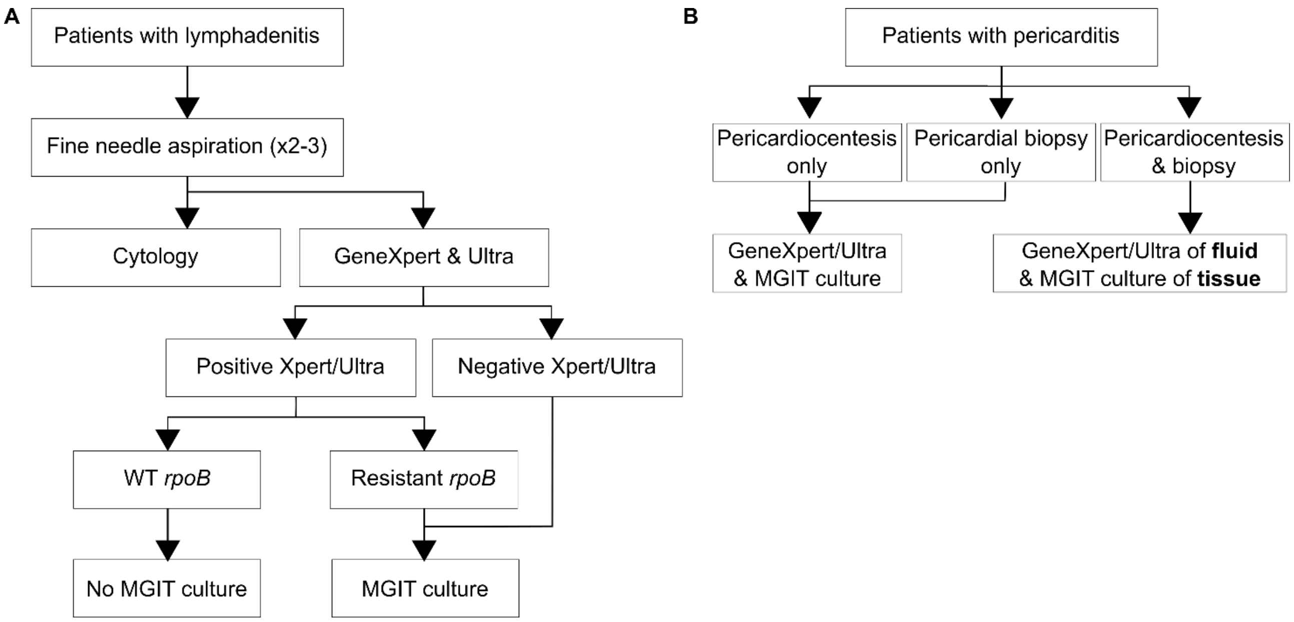

Testing algorithm for patients with possible TB lymphadenitis or pericarditis

(A) All patients under investigation for TB lymphadenitis had multipass fine needle aspiration. A sample for cytology was prioritised. The remaining sampling was used for PCR testing for Mtb using GeneXpert and Ultra. For those with negative PCR tests and those with positive PCR tests and rpoB mutations coferring rifampicin drug resistance any remaining sample was used for liquid culture using the Mycobacteria Growth Indicator Tube (MGIT960) platform. (B) All patients under investigation for TB pericarditis underwent sampling of pericardial fluid and or tissue biopsy. If only one sample type was collected, it was divided for both molecular testing and liquid culture. If both samples types were collected, the fluid was used for molecular testing and tissue biopsy was used for liquid culture.

Supplementary Figure 2

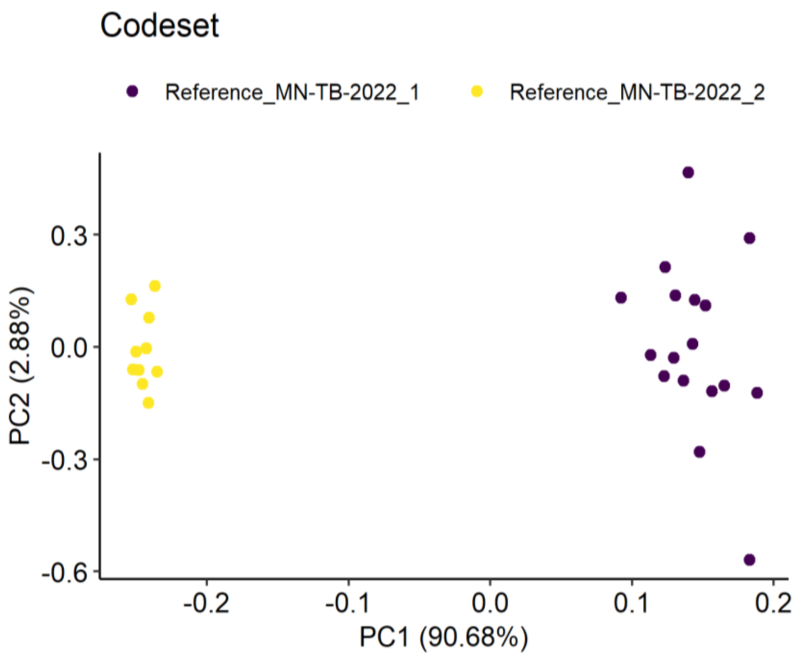

Principal component analysis of Nanostring results from reference RNA sample.

PCA clustering of standard reference RNA samples stratified by Nanostring codeset batch.

Supplementary Figure 3

Distribution of gene expression data stratified by Nanostring codeset batch

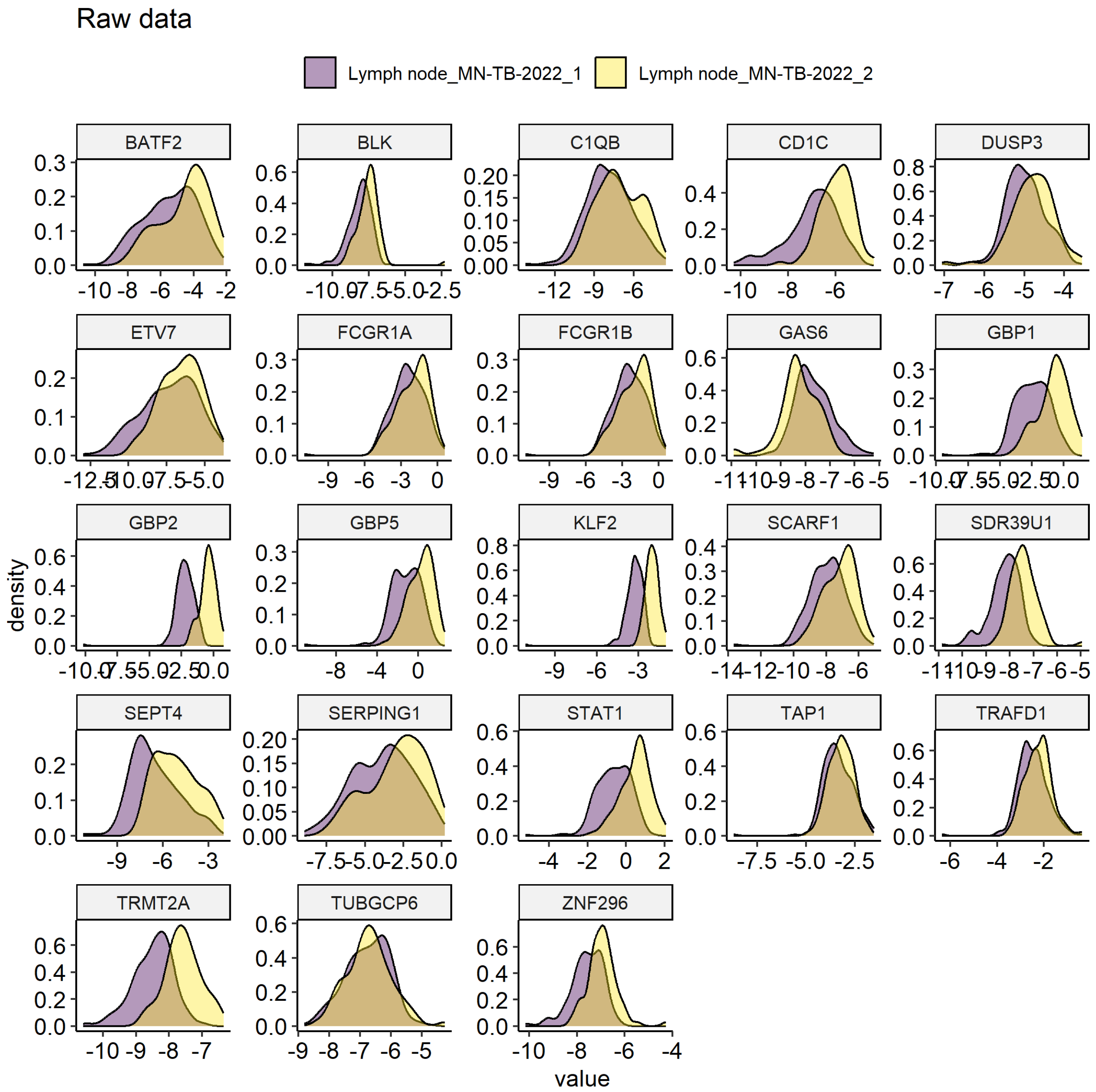

Gene level distributions of quantitative expression data in samples from lymphadenitis cohort stratified by Nanostring codeset batch.

Supplementary Figure 4

Distributions of TB genes after ComBat

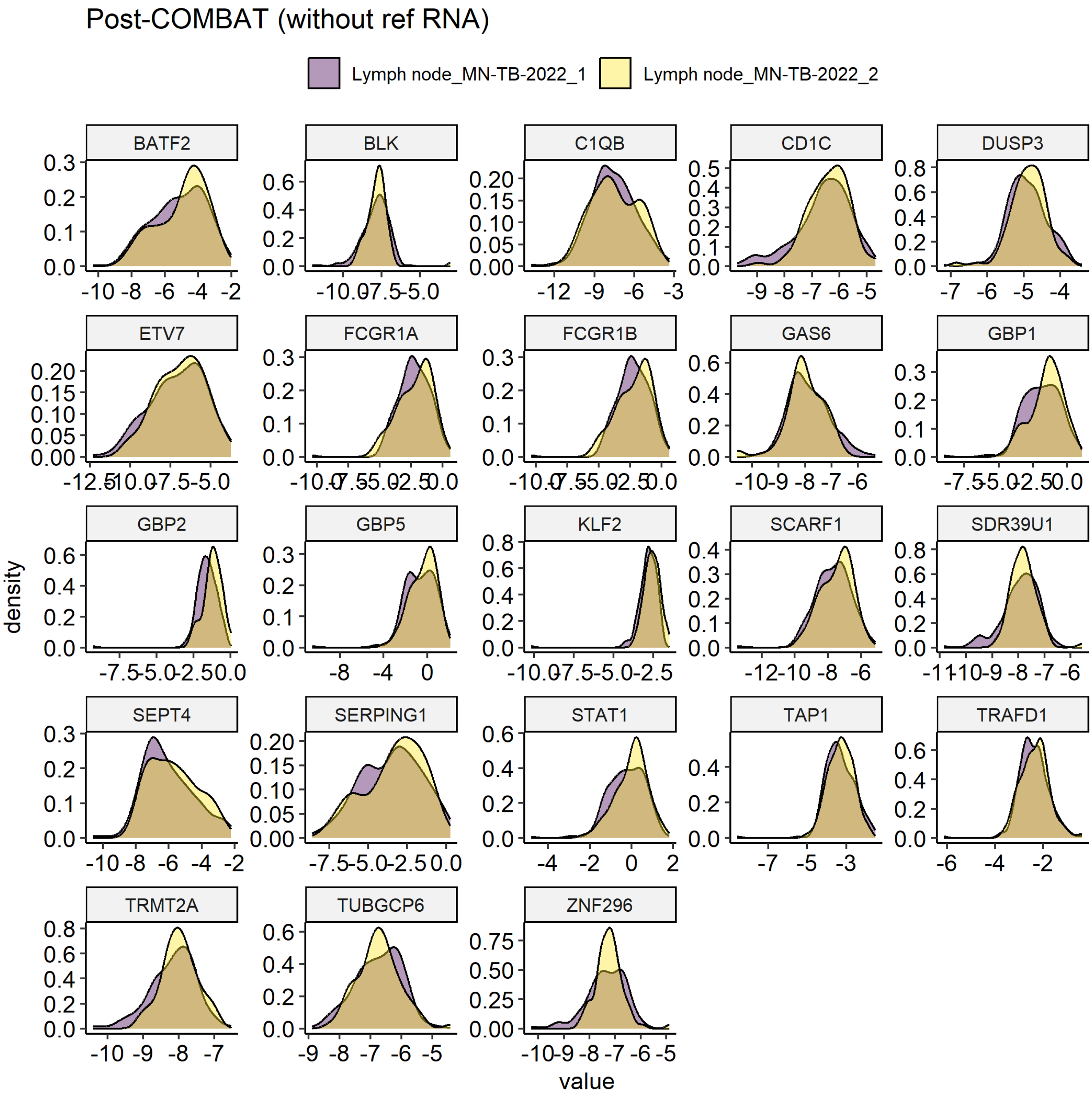

Gene level distributions of quantitative expression data in samples from lymphadenitis cohort stratified by Nanostring codeset batch following COMBAT batch correction.

Supplementary Figure 5

Sample size calculations.

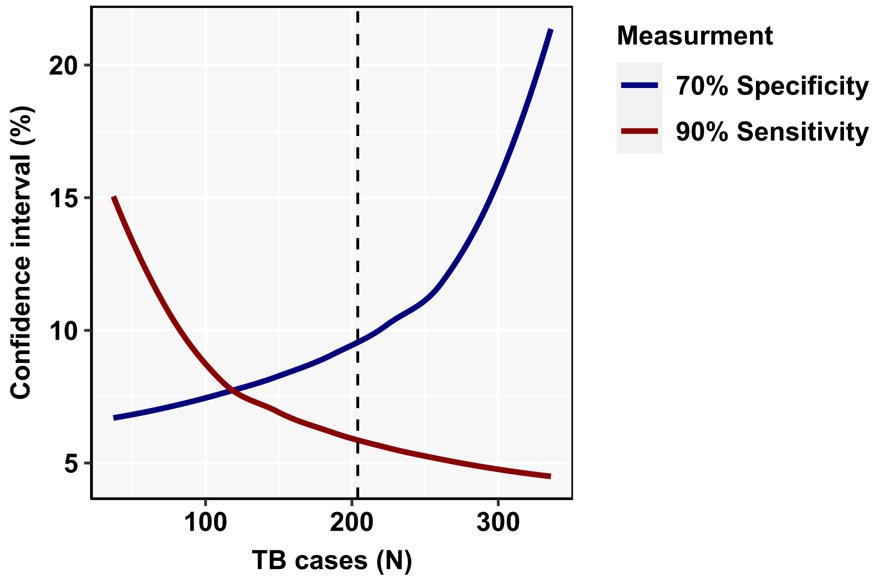

The relationship between confidence interval to detect a sensitivity of 90% and specificity of 70% is shown by number of TB cases for our total sample size (N=374) and power of >0.8, using PASS 2024 (Power Analysis and Sample Size Software, National Council for Social Studies). The dashed line shows the number of TB cases in our pooled data set (N=204).

Supplementary Figure 6

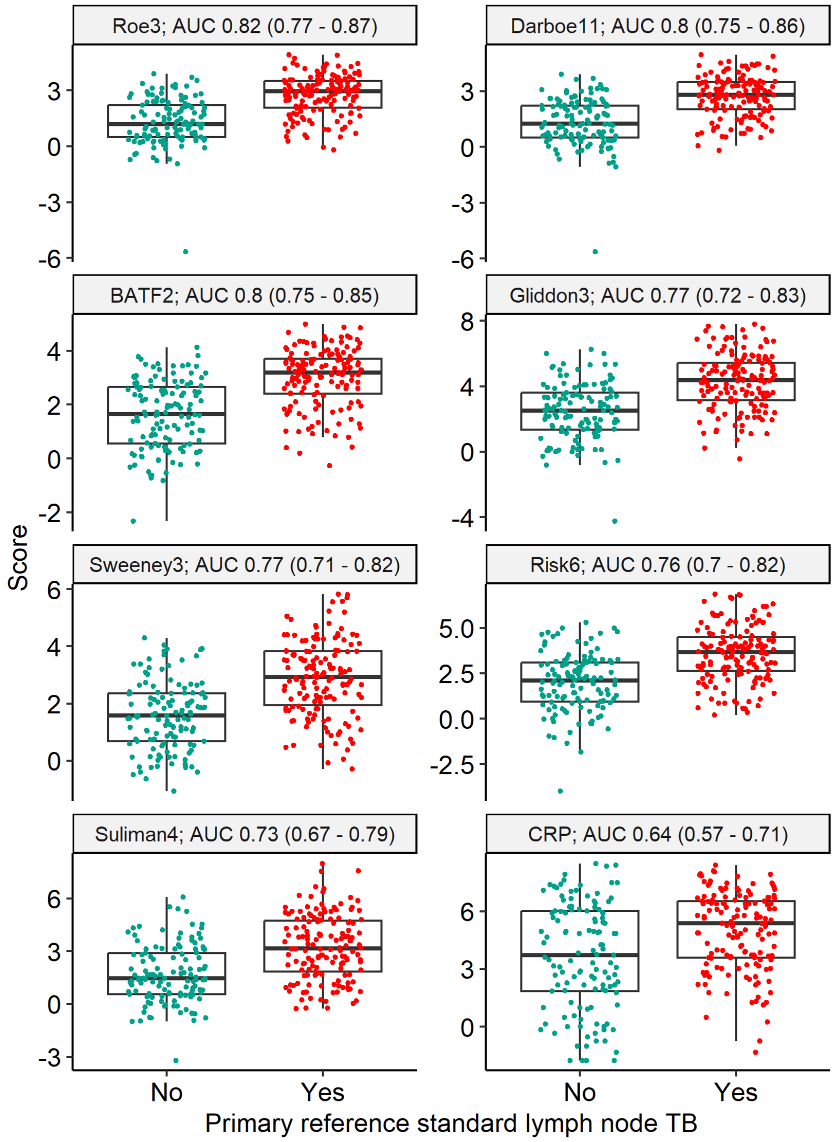

Blood RNA biomarker discrimination of primary reference standard for lymph node TB.

Scores are shown as Z-scores for RNA signatures, and log-2 transformed CRP (mg/L). Discrimination presented as area under the receiver operating characteristic curve (AUC), with 95% confidence intervals.

Supplementary Figure 7

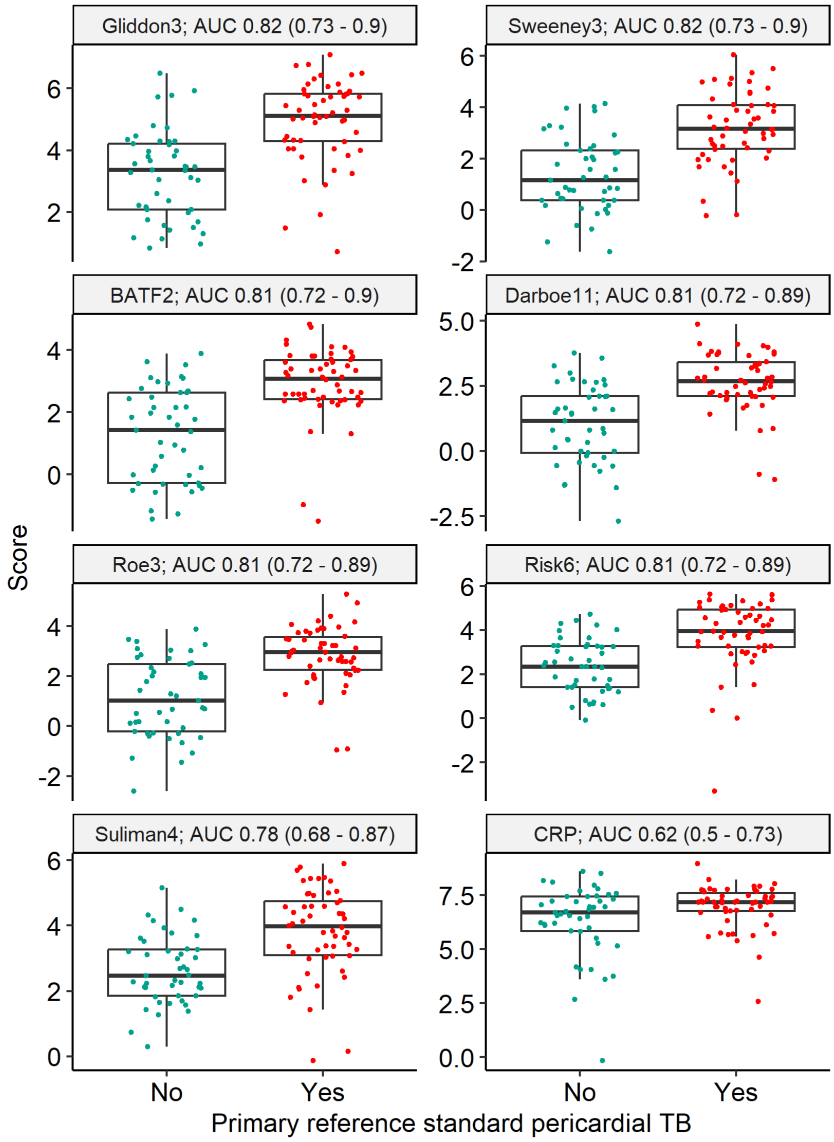

Blood RNA biomarker discrimination of primary reference standard for pericardial TB.

Scores are shown as Z-scores for RNA signatures, and log-2 transformed CRP (mg/L). Discrimination presented as area under the receiver operating characteristic curve (AUC), with 95% confidence intervals.

Supplementary Figure 8

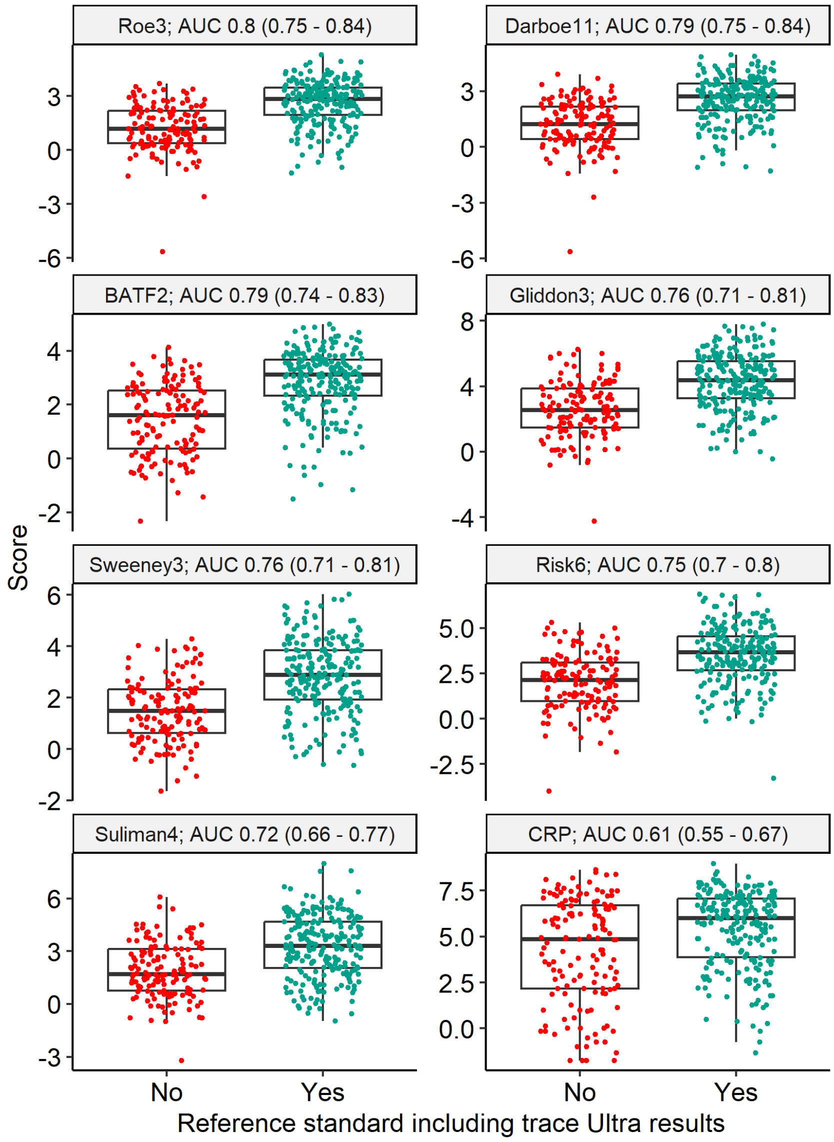

Blood RNA biomarker discrimination of lymph node and pericardial TB including trace Ultra results.

Scores are shown as Z-scores for RNA signatures, and log-2 transformed CRP (mg/L). Discrimination presented as area under the receiver operating characteristic curve (AUC), with 95% confidence intervals.

Supplementary Figure 9

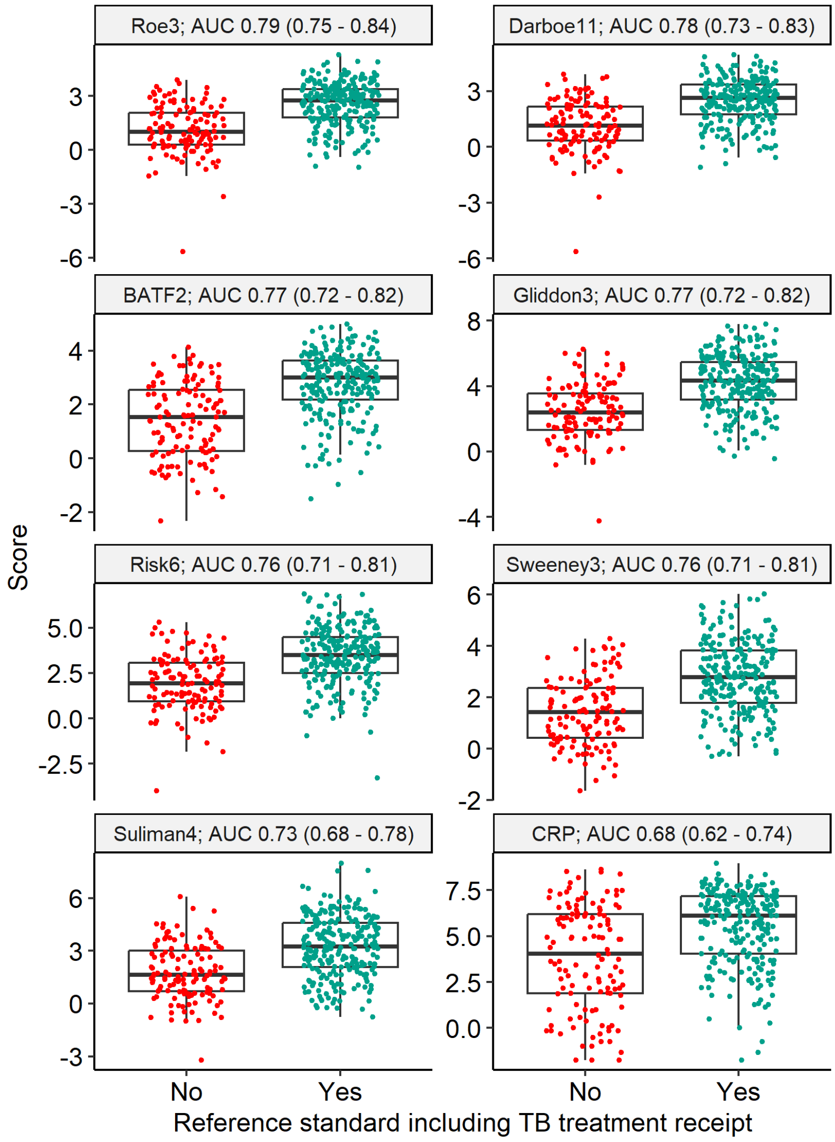

Blood RNA biomarker discrimination of lymph node and pericardial TB including TB treatment initiation.

Scores are shown as Z-scores for RNA signatures, and log-2 transformed CRP (mg/L). Discrimination presented as area under the receiver operating characteristic curve (AUC), with 95% confidence intervals.

Supplementary Figure 10

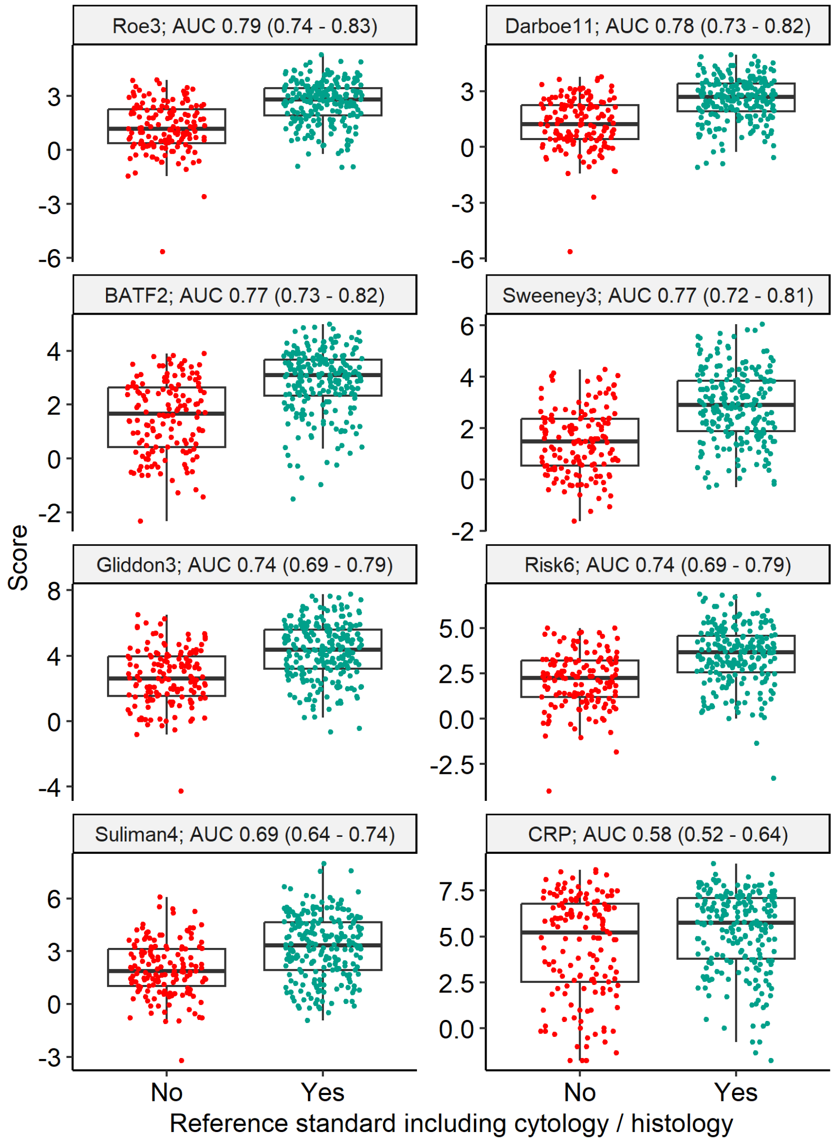

Blood RNA biomarker discrimination of lymph node and pericardial TB including including cytology and histology diagnosis of TB.

Scores are shown as Z-scores for RNA signatures, and log-2 transformed CRP (mg/L). Discrimination presented as area under the receiver operating characteristic curve (AUC), with 95% confidence intervals.

Supplementary Figure 11

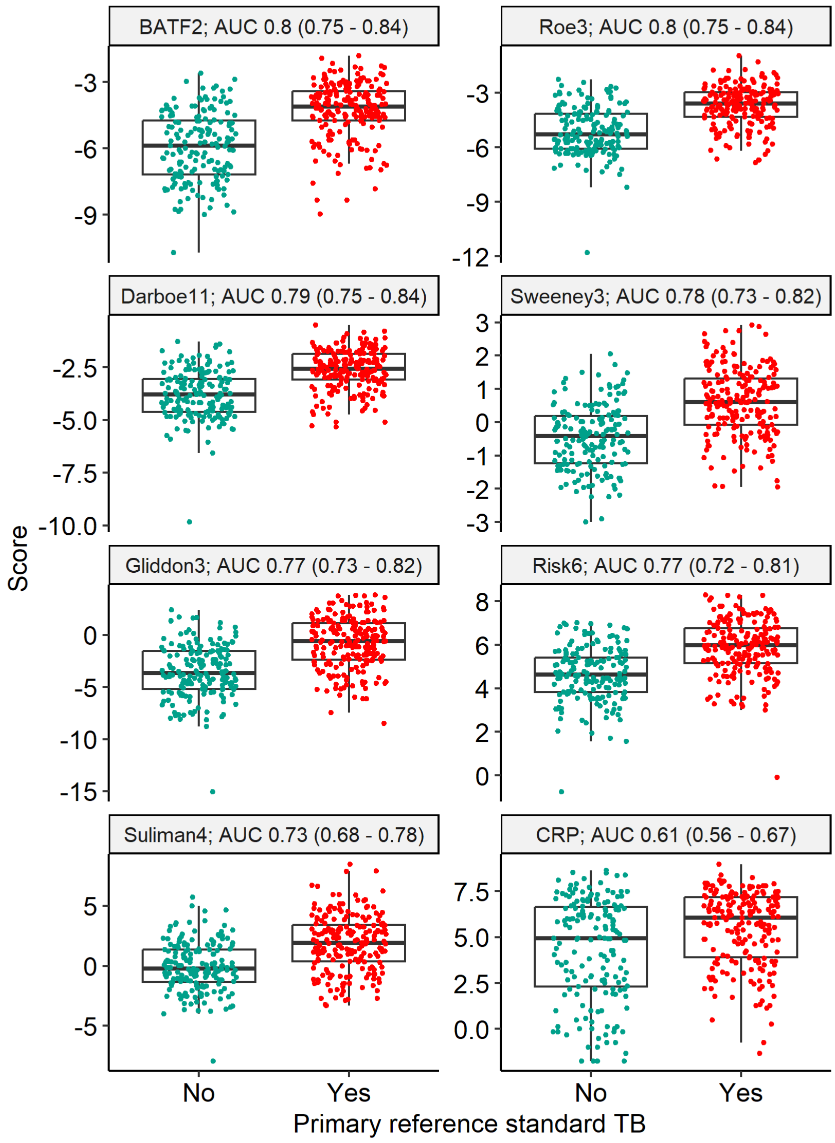

Blood RNA biomarker discrimination of primary reference standard for lymph node and pericardial TB using raw Nanostring data without batch correction.

Scores are shown as Z-scores for RNA signatures, and log-2 transformed CRP (mg/L). Discrimination presented as area under the receiver operating characteristic curve (AUC), with 95% confidence intervals.

Supplementary Table 1

Baseline characteristics of the study cohort, stratified by inclusion

**Characteristic**	**Overall**, N = 440[1]	**Included**, N = 374[1]	**Excluded**, N = 66[1]
Age (years)	38 (31, 48)	38 (31, 48)	39 (32, 49)
Gender			
Female	218 (50%)	181 (48%)	37 (56%)
Male	222 (50%)	193 (52%)	29 (44%)
Previous TB	110 (25%)	91 (24%)	19 (30%)
Missing	5	2	3
HIV status			
Negative	202 (46%)	172 (46%)	30 (47%)
Positive	234 (54%)	200 (54%)	34 (53%)
Missing	4	2	2
CD4 (cells/mm3)	157 (49, 287)	158 (49, 288)	154 (61, 275)
Missing	207	175	32
CD4 <200 cells/mm3	138 (59%)	118 (59%)	20 (59%)
Missing	207	175	32
On antiretroviral therapy	107 (47%)	95 (48%)	12 (38%)
Missing	210	176	34
Site			
Lymph node	300 (68%)	275 (74%)	25 (38%)
Pericardial	140 (32%)	99 (26%)	41 (62%)
Culture or molecular positive (site or sputum)	234 (53%)	204 (55%)	30 (47%)
Missing	2	0	2
Reason for exclusion			
No RNA data	58 (88%)	0 (NA%)	58 (88%)
No CRP data	6 (9.1%)	0 (NA%)	6 (9.1%)
No primary reference standard	2 (3.0%)	0 (NA%)	2 (3.0%)
Missing	374	374	0

[1]Median (IQR); n (%)

No systematic differences between included vs excluded participants. We included participants under investigation for presumptive lymph node or pericardial TB. Those with at least one molecular test result or TB culture from the site of disease, and CRP and RNA results available, were included.

Supplementary Table 2

Blood RNA biomarker and CRP performance metrics for discrimination of primary reference standard in lymph node and pericardial TB, using alternative cut-offs.

Test	Cutoff	Sensitivity	Specificity	PPV	NPV	Triage positive	NNT(+)	NNT(-)
CRP	5mg/L	0.92 (0.88-0.95)	0.25 (0.19-0.32)	0.6 (0.54-0.65)	0.73 (0.6-0.83)	0.84 (0.8-0.88)	1.7 (1.5-1.8)	3.7 (2.5-5.7)
BATF2	Maximal Youden Index	0.72 (0.65-0.77)	0.74 (0.66-0.8)	0.76 (0.7-0.82)	0.68 (0.61-0.75)	0.51 (0.46-0.56)	1.3 (1.2-1.4)	3.2 (2.6-3.9)
CRP	Maximal Youden Index	0.87 (0.82-0.91)	0.33 (0.26-0.4)	0.61 (0.55-0.66)	0.68 (0.58-0.77)	0.78 (0.74-0.82)	1.6 (1.5-1.8)	3.2 (2.4-4.4)
Gliddon3	Maximal Youden Index	0.6 (0.53-0.67)	0.84 (0.78-0.89)	0.82 (0.75-0.87)	0.64 (0.57-0.7)	0.4 (0.35-0.45)	1.2 (1.1-1.3)	2.8 (2.3-3.3)
Risk6	Maximal Youden Index	0.6 (0.53-0.66)	0.84 (0.78-0.89)	0.82 (0.75-0.87)	0.64 (0.57-0.7)	0.4 (0.35-0.45)	1.2 (1.1-1.3)	2.7 (2.3-3.3)
Roe3	Maximal Youden Index	0.71 (0.64-0.76)	0.79 (0.72-0.84)	0.8 (0.74-0.85)	0.69 (0.62-0.75)	0.48 (0.43-0.53)	1.2 (1.2-1.4)	3.2 (2.6-4)
Suliman4	Maximal Youden Index	0.75 (0.68-0.8)	0.62 (0.54-0.69)	0.7 (0.64-0.76)	0.67 (0.59-0.74)	0.58 (0.53-0.63)	1.4 (1.3-1.6)	3 (2.5-3.8)
Sweeney3	Maximal Youden Index	0.67 (0.6-0.73)	0.79 (0.72-0.84)	0.79 (0.73-0.85)	0.67 (0.6-0.73)	0.46 (0.41-0.51)	1.3 (1.2-1.4)	3 (2.5-3.7)
Darboe11	Maximal Youden Index	0.8 (0.74-0.85)	0.65 (0.58-0.72)	0.74 (0.67-0.79)	0.74 (0.66-0.8)	0.6 (0.55-0.64)	1.4 (1.3-1.5)	3.8 (2.9-5)
BATF2	Sensitivity 90%	0.9 (0.85-0.93)	0.44 (0.37-0.51)	0.66 (0.6-0.71)	0.78 (0.69-0.86)	0.75 (0.7-0.79)	1.5 (1.4-1.7)	4.6 (3.2-6.9)
CRP	Sensitivity 90%	0.9 (0.85-0.93)	0.27 (0.21-0.34)	0.6 (0.54-0.65)	0.69 (0.57-0.79)	0.82 (0.78-0.86)	1.7 (1.5-1.8)	3.3 (2.4-4.8)
Gliddon3	Sensitivity 90%	0.9 (0.85-0.93)	0.39 (0.32-0.47)	0.64 (0.58-0.69)	0.77 (0.67-0.84)	0.77 (0.72-0.81)	1.6 (1.4-1.7)	4.3 (3-6.4)
Risk6	Sensitivity 90%	0.9 (0.85-0.93)	0.38 (0.31-0.45)	0.63 (0.58-0.69)	0.76 (0.66-0.84)	0.77 (0.73-0.81)	1.6 (1.5-1.7)	4.1 (2.9-6.1)
Roe3	Sensitivity 90%	0.9 (0.85-0.93)	0.52 (0.45-0.6)	0.69 (0.64-0.75)	0.81 (0.73-0.88)	0.71 (0.66-0.75)	1.4 (1.3-1.6)	5.4 (3.7-8)
Suliman4	Sensitivity 90%	0.9 (0.85-0.93)	0.27 (0.21-0.34)	0.6 (0.54-0.65)	0.69 (0.57-0.79)	0.82 (0.78-0.86)	1.7 (1.5-1.8)	3.3 (2.3-4.8)
Sweeney3	Sensitivity 90%	0.9 (0.85-0.93)	0.42 (0.35-0.5)	0.65 (0.59-0.71)	0.78 (0.68-0.85)	0.75 (0.71-0.79)	1.5 (1.4-1.7)	4.5 (3.2-6.7)
Darboe11	Sensitivity 90%	0.9 (0.85-0.93)	0.45 (0.37-0.52)	0.66 (0.6-0.71)	0.79 (0.7-0.86)	0.74 (0.7-0.78)	1.5 (1.4-1.7)	4.7 (3.3-7)

Supplementary Table 3

Blood RNA biomarker and CRP performance metrics for discrimination of primary reference standard in lymph node TB.

Signature	AUROC	Sensitivity	Specificity	PPV	NPV	Triage positive	NNT(+)	NNT(-)	p
Roe3	0.82 (0.77-0.87)	0.76 (0.69-0.82)	0.7 (0.61-0.77)	0.75 (0.68-0.81)	0.71 (0.62-0.78)	0.55 (0.49-0.61)	1.3 (1.2-1.5)	3.4 (2.6-4.6)	p<0.0001
Darboe11	0.8 (0.75-0.86)	0.75 (0.68-0.82)	0.66 (0.58-0.74)	0.73 (0.65-0.79)	0.69 (0.6-0.77)	0.56 (0.5-0.62)	1.4 (1.3-1.5)	3.2 (2.5-4.3)	p<0.0001
BATF2	0.8 (0.75-0.85)	0.79 (0.72-0.85)	0.58 (0.49-0.66)	0.69 (0.62-0.76)	0.7 (0.6-0.78)	0.63 (0.57-0.68)	1.4 (1.3-1.6)	3.3 (2.5-4.5)	p<0.0001
Gliddon3	0.77 (0.72-0.83)	0.91 (0.85-0.94)	0.42 (0.33-0.5)	0.65 (0.58-0.71)	0.79 (0.67-0.87)	0.76 (0.71-0.81)	1.5 (1.4-1.7)	4.7 (3.1-7.6)	p<0.0001
Sweeney3	0.77 (0.71-0.82)	0.75 (0.67-0.81)	0.65 (0.56-0.73)	0.72 (0.64-0.78)	0.68 (0.59-0.76)	0.57 (0.51-0.62)	1.4 (1.3-1.6)	3.1 (2.5-4.1)	p<0.0001
Risk6	0.76 (0.7-0.82)	0.85 (0.78-0.9)	0.46 (0.38-0.55)	0.65 (0.59-0.72)	0.72 (0.61-0.8)	0.71 (0.65-0.76)	1.5 (1.4-1.7)	3.5 (2.6-5.1)	p<0.0001
Suliman4	0.73 (0.67-0.79)	0.73 (0.65-0.79)	0.64 (0.55-0.72)	0.71 (0.63-0.77)	0.66 (0.57-0.74)	0.56 (0.5-0.62)	1.4 (1.3-1.6)	3 (2.3-3.8)	0.002
CRP	0.64 (0.57-0.71)	0.77 (0.7-0.83)	0.46 (0.38-0.55)	0.63 (0.56-0.7)	0.63 (0.53-0.72)	0.67 (0.61-0.72)	1.6 (1.4-1.8)	2.7 (2.1-3.6)	

P values indicate pairwise comparisons to CRP, with multiple testing correction.

Supplementary Table 4

Blood RNA biomarker and CRP performance metrics for discrimination of primary reference standard in pericardial TB.

Signature	AUROC	Sensitivity	Specificity	PPV	NPV	Triage positive	NNT(+)	NNT(-)	p
Gliddon3	0.82 (0.73-0.9)	0.94 (0.85-0.98)	0.24 (0.14-0.39)	0.6 (0.49-0.7)	0.79 (0.52-0.92)	0.86 (0.78-0.91)	1.7 (1.4-2)	4.7 (2.1-13.2)	0.012
Sweeney3	0.82 (0.73-0.9)	0.81 (0.69-0.9)	0.64 (0.5-0.77)	0.73 (0.61-0.83)	0.74 (0.59-0.85)	0.61 (0.51-0.7)	1.4 (1.2-1.6)	3.9 (2.4-6.9)	0.012
BATF2	0.81 (0.72-0.9)	0.93 (0.82-0.97)	0.62 (0.48-0.75)	0.75 (0.63-0.84)	0.88 (0.72-0.95)	0.68 (0.58-0.76)	1.3 (1.2-1.6)	8 (3.6-20.1)	0.016
Risk6	0.81 (0.72-0.89)	0.91 (0.8-0.96)	0.42 (0.29-0.57)	0.65 (0.54-0.75)	0.79 (0.6-0.91)	0.76 (0.66-0.83)	1.5 (1.3-1.8)	4.8 (2.5-10.8)	0.016
Roe3	0.81 (0.72-0.89)	0.83 (0.71-0.91)	0.67 (0.52-0.79)	0.75 (0.63-0.84)	0.77 (0.62-0.87)	0.61 (0.51-0.7)	1.3 (1.2-1.6)	4.3 (2.6-7.9)	0.016
Darboe11	0.81 (0.72-0.89)	0.83 (0.71-0.91)	0.67 (0.52-0.79)	0.75 (0.63-0.84)	0.77 (0.62-0.87)	0.61 (0.51-0.7)	1.3 (1.2-1.6)	4.3 (2.6-7.9)	0.016
Suliman4	0.78 (0.68-0.87)	0.93 (0.82-0.97)	0.27 (0.16-0.41)	0.6 (0.49-0.7)	0.75 (0.51-0.9)	0.84 (0.75-0.9)	1.7 (1.4-2)	4 (2-9.8)	0.016
CRP	0.62 (0.5-0.73)	0.98 (0.9-1)	0.04 (0.01-0.15)	0.55 (0.45-0.65)	0.67 (0.21-0.98)	0.97 (0.91-0.99)	1.8 (1.5-2.2)	3 (1.3-58.5)	

P values indicate pairwise comparisons to CRP, with multiple testing correction.

Supplementary Table 5

Blood RNA biomarker and CRP performance metrics for discrimination of TB status, stratified by selected subgroups.

Signature	HIV-positive	HIV-negative	p
BATF2	0.8 (0.74-0.86)	0.79 (0.73-0.86)	0.954
CRP	0.62 (0.54-0.71)	0.57 (0.48-0.65)	0.846
Gliddon3	0.78 (0.72-0.84)	0.75 (0.68-0.83)	0.926
Risk6	0.78 (0.72-0.84)	0.73 (0.66-0.81)	0.846
Roe3	0.81 (0.75-0.87)	0.8 (0.74-0.87)	0.954
Suliman4	0.77 (0.7-0.83)	0.65 (0.57-0.73)	0.238
Sweeney3	0.79 (0.73-0.86)	0.75 (0.68-0.83)	0.846
Darboe11	0.8 (0.74-0.86)	0.8 (0.73-0.87)	0.954
Signature	On ART	No ART	p
BATF2	0.74 (0.64-0.84)	0.84 (0.76-0.91)	0.185
CRP	0.56 (0.44-0.68)	0.64 (0.51-0.77)	0.381
Gliddon3	0.69 (0.58-0.8)	0.84 (0.76-0.92)	0.084
Risk6	0.69 (0.59-0.8)	0.84 (0.76-0.92)	0.084
Roe3	0.77 (0.67-0.86)	0.84 (0.77-0.92)	0.24
Suliman4	0.67 (0.56-0.78)	0.85 (0.77-0.92)	0.084
Sweeney3	0.72 (0.62-0.82)	0.85 (0.78-0.93)	0.084
Darboe11	0.74 (0.64-0.84)	0.84 (0.76-0.91)	0.185

Accuracy shown for primary reference standard as area under the receiver operating characteristic curve with 95% confidence intervals. P values indicate comparisons between strata for each signature.
